# Surfactant replacement therapy in late preterm respiratory disease: Identifying suitable candidates

**DOI:** 10.1038/s41390-025-04705-7

**Published:** 2025-12-17

**Authors:** Eleanor Jeffreys, Theodore Dassios

**Affiliations:** 1 Neonatal Intensive Care Unit, King’s College Hospital NHS Foundation Trust, London, UK; 2 Department of Women and Children’s Health, Faculty of Life Sciences and Medicine, King’s College London, London, UK; 3 Neonatal Intensive Care Unit, University of Patras, Patras, Greece

Late preterm infants are defined as those born between 34 and 37 weeks of gestation and constitute the majority of all premature births. Although late preterms are a lower-risk group compared to more immature preterm infants, they suffer from significant respiratory morbidity and exert considerable pressure on neonatal services, because of their sheer number and frequent admission for neonatal care.^1^ Late preterm infants might suffer from isolated respiratory distress syndrome due to lack of endogenous surfactant and lung immaturity, or often they might exhibit a mixed phenotype which is the result of a combination of lung immaturity and concomitant respiratory morbidities.

In this issue of Paediatric Research, Sadowska-Krawczenko and co-authors evaluated the effectiveness of administering exogenous surfactant in a multicenter prospective study of 350 late preterm infants by measuring the improvement in oxygenation, estimated by the saturation to fraction of inspired oxygen ratio (SFR). They reported that six hours after surfactant delivery, 64% of the included infants had an improvement in the SFR of more than 50%, with a larger improvement in oxygenation when the exogenous surfactant was administered earlier. Late preterms with isolated respiratory distress syndrome were more likely to respond well to exogenous surfactant while comorbidities such as pneumonia, air leaks, pulmonary haemorrhage, pulmonary hypertension of the newborn and meconium aspiration syndrome were associated with a more limited response to exogenous surfactant or no response at all.^2^ These are very useful observations as they highlight the efficiency of administering exogenous surfactant in this relatively large population of infants, while they also underpin that surfactant will act better when given early, similar to what has been previously well-described in more immature preterms below 32 weeks of gestation.^3^

It is interesting to consider the definition of a “*good response to surfactant*” employed by the authors as a 50% improvement in the SFR, or an SFR of more than 428 recorded at six hours after surfactant, which roughly translates to a decrease of the oxygen requirement from 40% to 27% while the saturation remains 90%.^2^ It would, thus, follow that the group of the “*non-responders*” might also contain a significant number of late preterms who did respond by an improvement of oxygenation with an increase in the SFR of less than 50%, and according to the study these are mostly the infants with coexisting pulmonary morbidities. The *non-responders* had a mean improvement in the SFR at 24 hours of 94.4 with a standard deviation of 81.6, which means that the majority of the “non-responders” also had a positive response to surfactant (improvement in oxygenation), albeit of a lower magnitude than the predefined threshold of 50%. In reality, only a very small fraction of the presented infants had a neutral or negative change in oxygenation in response to surfactant.

Additionally, the non-responders received surfactant approximately eight hours (difference of the median age at surfactant administration) later than the responders and had lower oxygen requirements when surfactant was given. Possibly the natural progression of the disease is such that oxygen requirements decrease over time as the lungs recover and any additional fluid is expelled, but this might happen less quickly than if surfactant had been given. It could be, thus, postulated that the non-responders had higher oxygen requirements earlier on, for example, at a time point similar to the time the responders received surfactant, and that a larger drop in oxygen requirements and therefore an increase in SFR would have been seen if surfactant had been administered earlier.

It is commendable that nearly 80% of the included infants had a lung ultrasound prior to surfactant administration. The widespread use of ultrasound in the participating neonatal units is a positive development, as it pairs well with the observation that early surfactant administration is beneficial, and this early administration can be achieved by avoiding delays related to timing and availability of chest radiography. Some epidemiological observations regarding the study population might bear implications for the generalizability of the study results. The authors report an overall rate of caesarean section of 75%. Recently, a study that included the majority of the European countries reported that in the period between 1999 and 2023, Poland had the highest rate of caesarean section (55%), while other countries had considerably lower rates (for example, France had 19%).^4^ Delivery via caesarean section might impact the major outcome of the study, which is the need for surfactant and might introduce an unknown element of delayed lung fluid clearance, which might impact the severity of respiratory disease in late preterm infants. Caesarean delivery is known to be associated with a higher risk of respiratory complications in the offspring (such as transient tachypnoea and respiratory distress syndrome), possibly via an absence of hormonal signals and stress-related lung maturation and by the lack of vaginal delivery during which some beneficial pressure is exerted on the chest during the passage through the birth canal.^5^

Another potentially important area where this study population might differ from the general late preterm population across the world is the relatively low utilisation of high-flow nasal cannula therapy. The authors reported that only 0.3% of the included infants were treated with high-flow, and the majority of infants on non-invasive support were treated with either plain continuous positive airways pressure (29%), biphasic positive airway pressure (25%) or nasal intermittent positive pressure ventilation (22%).^2^ This is considerably lower compared to what has been reported in other populations. In the United States, the Vermont Oxford Network reported that the use of high-flow nasal cannulae in infants born below 1500 g was 58% in 2009,^6^ while a nationwide survey published in 2013 showed that high-flow was already in use in 77% of the tertiary neonatal units in the United Kingdom.^7^ This is potentially an important point, as the pathophysiology of respiratory disease in late preterm infants might make high-flow particularly useful for this population, as it has been demonstrated that failure of high-flow is associated with a lower gestational age of the treated infants.^8^ It is plausible that although very and extremely preterm infants are distinctly reliant on the provision of a significant distending alveolar pressure, which is administered to prevent atelectasis and alveolar collapse, late preterms might be naturally less dependent on this mechanism due to their advanced morphological lung maturity.

The actual impact of these specific population particularities on the efficiency of surfactant in late preterm infants with respiratory disease might be clarified in the near future with the publication of the SurfON multicenter randomized controlled trial, which recruited more than 1500 late preterm and early term infants (34 to 38 weeks of gestational age) from 45 neonatal units in the United Kingdom and investigated whether early administration of surfactant, when compared to expectant management, reduced the length of neonatal hospital stay and reduced the progression to more severe respiratory illness.^9^ It is notable that the SurfON study also included longer-term clinical outcomes such as the duration of respiratory support and duration of hospital stay, as well as health economic outcomes relating to the cost of stay and the effect on the maternal quality of life, which were not intended to be captured by the study of Sadowska-Krawczenko and co-workers.

The strength of the conclusions regarding the lack of effectiveness of surfactant for some rare outcomes, such as pulmonary haemorrhage should also be interpreted with some caution, as the total incidence of pulmonary haemorrhage in the whole population was 2%, with only two and five events in the two categories of responders and non-responder,s respectively. From a purist perspective, there might be a slightly loose interpretation of the definition of the population as *late preterm*, as the mean gestational age of all included infants was 35.8 weeks with a standard deviation of 1.6 weeks, which based on the 68–95–99.7 rule, implies that approximately 15% of the population would be more than 37 weeks, so technically not preterm. This however, should not decrease the validity of the study conclusions, since late preterm and early term infants are frequently grouped together for purposes of quantifying respiratory morbidity as seen in the aforementioned SurfON study.^9^

In conclusion, the study of Sadowska-Krawczenko and co-authors highlighted that surfactant replacement was useful and effective in the great majority of late preterm infants with respiratory distress, while early administration was associated with a better response. Within the group of late preterm infants, the ones with a lower gestation, the ones who needed a high concentration of oxygen and had isolated surfactant deficiency in the absence of other pulmonary comorbidities appeared to have benefited more from treatment with surfactant.
